# Identification of risk factors in myocardial infarction with non-obstructive coronary arteries

**DOI:** 10.1590/1806-9282.20242040

**Published:** 2025-07-07

**Authors:** Aydın Dursun, Hakan Guven, Mehmet Cem Basel, Mustafa Selcuk Atasoy, Ahmet Yuksel

**Affiliations:** 1 Mudanya University, VM Medical Park Hospital, Department of Cardiology – Bursa, Türkiye.; 2 Mudanya University, VM Medical Park Hospital, Department of Cardiovascular Surgery – Bursa, Türkiye.; 3 University of Health Sciences, Bursa City Hospital, Bursa Faculty of Medicine, Department of Cardiovascular Surgery – Bursa, Türkiye.

**Keywords:** Myocardial infarction, Coronary artery disease, Acute coronary syndrome, Risk factors, Vitamin D

## Abstract

**OBJECTIVE:**

The aim of this study was to identify the risk factors and potential causes of myocardial infarction with non-obstructive coronary arteries in patients presenting with acute coronary syndrome.

**METHODS:**

This retrospective case series study was conducted at the coronary intensive care unit of our hospital between January 2019 and December 2023. Of 3,107 acute coronary syndrome patients, 195 diagnosed with myocardial infarction with non-obstructive coronary arteries were included. Clinical characteristics, risk factors, laboratory test results, and electrocardiographic, echocardiographic, and coronary angiography findings were analyzed. Additionally, vitamin D, homocysteine, folate, and vitamin B12 levels were compared between myocardial infarction with non-obstructive coronary arteries patients and controls with non-occlusive coronary artery disease without acute coronary syndrome.

**RESULTS:**

Among a total of 3,107 acute coronary syndrome patients, 195 (6.28%) were diagnosed with myocardial infarction with non-obstructive coronary arteries. Their mean age was 53.9 years, and 83% of the patients were male. Hypertension (45%), diabetes mellitus (33%), and smoking (70%) were prominent risk factors. Myocardial bridge was the most common cause of myocardial infarction with non-obstructive coronary arteries (11.2%). The left anterior descending artery was the most frequent single vessel responsible for acute coronary syndrome (26%). Vitamin D, B12, and folate levels were statistically significantly lower in myocardial infarction with non-obstructive coronary arteries patients compared to controls (p<0.05).

**CONCLUSIONS:**

Smoking and hypertension were prominent risk factors for myocardial infarction with non-obstructive coronary arteries. Myocardial bridge was the most commonly identified cause, and vitamin D deficiency was significantly associated with myocardial infarction with non-obstructive coronary arteries.

## INTRODUCTION

Myocardial infarction with non-obstructive coronary arteries (MINOCA) is a clinical condition that has received increasing attention in recent years but is still not fully understood^1^. This condition is diagnosed in patients with classic myocardial infarction (MI) symptoms but without significant stenosis (<50%) detected on coronary angiography^1^.

The pathogenesis of MINOCA is based on highly heterogeneous causes, and it is understood that traditional MI treatment strategies do not always yield optimal results in these patients^2^. Although studies on the diagnostic and therapeutic approaches of MINOCA are increasing, the management of this patient group still contains some uncertainties^3^. The epidemiology and clinical features of MINOCA differ from obstructive CAD, as it is more common in younger patients and women, with a lower rate of hyperlipidemia^4^. However, no significant differences exist in other traditional risk factors^4^. These distinctions necessitate careful consideration of management and treatment strategies for MINOCA patients. The diagnosis of MINOCA is considered a "working diagnosis" after coronary angiography, and further examinations are essential to determine whether another pathology is causing ischemic or non-ischemic myocardial damage^5^. Cardiac magnetic resonance imaging (CMRI) plays a critical role in confirming the diagnosis of MINOCA, and current clinical guidelines strongly recommend its use^1^. However, there is a great inconsistency in the diagnostic processes due to the differences in practice and financial constraints in different centers^6^. As a result, the etiology cannot be determined in approximately 25% of MINOCA patients despite all evaluations^7^.

Although the prognosis of MINOCA is generally reported to be better than obstructive CAD, this condition does not always follow a benign course^8^. Mortality rates have been found to be higher in MINOCA patients compared to healthy individuals^3^.

Studies on the long-term clinical outcomes of MINOCA have shown that patients face serious events such as cardiovascular death, re-infarction, and heart failure hospitalization within 1 year, highlighting the importance of early recognition and accurate determination of the underlying pathophysiology for appropriate treatment strategies^9^. A better understanding of the pathophysiological mechanisms underlying the disease will provide significant progress in the management of this patient group.

In our study, we aimed to determine the risk factors and potential causes of MINOCA in patients presenting with acute coronary syndrome (ACS), and to define this disease group more clearly in the future, and to develop personalized treatment strategies.

## METHODS

### Study design and population

This retrospective study, conducted at the Coronary Care Unit of Bursa VM MediKal Park Hospital between January 2019 and December 2023, was approved by the Mudanya University's Research Ethics Committee (decision number 2024/10/01/05).

A total of 3,107 patients admitted with ACS were screened, and 195 patients (6.28%) diagnosed with MINOCA were included.

### Inclusion criteria

Patients admitted with ACS [angina at rest, suggestive electrocardiographic (ECG) changes, and elevated troponin I levels].

Non-obstructive coronary arteries (<50% stenosis) on coronary angiography.

### Exclusion criteria

- Patients with significant coronary artery stenosis (≥50%).
- Clinically overt non-cardiac causes of elevated troponin (e.g., myocarditis, pulmonary embolism).

### Data collection

Data were collected retrospectively from medical records:

- Clinical characteristics: Age, gender, a history of hypertension, diabetes mellitus, smoking, and a family history of coronary artery disease (CAD).
- Laboratory parameters: Vitamin D, B12, folate, homocysteine, lipid profile, C-reactive protein (CRP), N-terminal pro-B-type natriuretic peptide (NT-proBNP), troponin I, and complete blood count.

3. Imaging findings:
  - Coronary angiography: Identification of plaques (<30%) and mild stenosis (30–50%).
  - Echocardiography: Left ventricular wall motion abnormalities and ejection fraction using Simpson's method.

### Statistical analysis

Statistical analyses were performed using MedCalc (MedCalc, Mariakerke, Belgium) software. Continuous variables were expressed as mean±standard deviation, and categorical variables were expressed as percentages. Comparisons between groups were made using the Student's t-test and the Mann-Whitney U test. A p<0.05 was considered statistically significant.

## RESULTS

### Baseline characteristics (Table 1)

**Table 1:** Baseline characteristics and laboratory features of the patients.

Variables	MINOCA (n=195)	Control (n=180)	p-value
Age (years)	54.4±12.9	57.1±12.6	0.3208
Male gender, n (%)	37 (74%)	32 (64%)	0.3871
Diabetes mellitus, n (%)	18 (36%)	20 (40%)	0.8368
Smoking, n (%)	30 (60%)	27 (54%)	0.6862
Hypertension, n (%)	22 (44%)	25 (50%)	0.6886
25-OH Vitamin D (ng/mL)	11.58±1.55	33.29±3.28	<0.001*
Homocysteine (μmol/L)	19.90±4.68	15.01±1.82	0.443
Vitamin B12 (pg/mL)	269.3±26.9	375.7±40.3	0.062*
Folate (ng/mL)	5.27±0.73	7.73±0.37	0.006*
NT-Pro BNP (pg/mL)	2,190.6±29.6	333.3±16.5	0.207
CRP (mg/L)	28.2±9.7	3.89±0.84	0.006*
Troponin I	2,315±56.11	4.05±0.69	0.020*
LDL (mg/dL)	112.4±5.45	111.2±6.42	0.884
White blood cell (WBC) count	9.4±3.0	7.7±2.1	0.003*
WBC/PDW	0.89±0.2	0.76±0.2	0.026*
Neu/Lenf ratio	6.75±2.27	2.19±0.12	0.050*
Platelet distribution width	14.81±2.34	16.14±0.31	0.005*

MINOCA: myocardial infarction with non-obstructive coronary arteries; NT-Pro BNP: N-terminal pro-B-type natriuretic peptide; CRP: C-reactive protein; WBC: white blood cell; LDL: low density lipoprotein; PDW: platelet distribution width; MVR: mitral valve replacement; LAD-RCA: left anterior descending-right coronary artery; LAD-LCX: left anterior descending-left circumflex; LAD-LCX-RCA: left anterior descending-left circumflex -right coronary artery.

* Statistically significant. Neu/Lenf ratio=neutrophil/lymphocyte ratio.

Among a total of 3,107 ACS patients, 195 (6.28%) were diagnosed with MINOCA. Their mean age was 53.9±12.9 years, and 83% of the patients were male. Hypertension was present in 45% of the patients, diabetes mellitus in 33%, and smoking in 70%.

### Laboratory findings (Table 1)

- Vitamin D levels were statistically significantly lower in MINOCA patients (11.58±1.55 ng/mL) compared to controls (33.29±3.28 ng/mL, p<0.001).
- Folate levels were also lower in the MINOCA group (p=0.006).
- Troponin I levels were statistically significantly elevated in MINOCA patients compared to controls (p=0.020).

### Etiology and angiographic findings (Tables 2 and 3)

**Table 2:** Causes of acute coronary syndrome.

Causes	MINOCA (n=195)	Control (n=180)	p-value
Coronary slow flow phenomenon, n (%)	18 (9.2%)	15 (8.3%)	0.9004
Coronary dissection, n (%)	1 (0.5%)	0 (0%)	0.9482
Myocardial bridge (MB), n (%)	22 (11.2%)	17 (9.4%)	0.6875
Coronary artery ectasia, n (%)	7 (3.58%)	3 (1.6%)	0.382
Hypertrophic/dilated cardiomyopathy	2 (1.0%)	1 (0.5%)	0.9696
Infection (e.g., cholangitis, pneumonia)	5 (2.56%)	1 (0.5%)	0.2352
Previous valve surgery (MVR), n (%)	3 (1.5%)	0 (0%)	0.2882
Coronary plaque (<50% stenosis), n (%)	125 (64%)	140 (77%)	0.0083*
Atrial fibrillation, n (%)	7 (3.6%)	1 (0.5%)	0.0843

MINOCA: myocardial infarction with non-obstructive coronary arteries.

* Statistically significant.

**Table 3:** Distribution of ACS-responsible lesions in MINOCA.

Coronary lesion	MINOCA (n=195)	Control (n=180)	p-value
Left anterior descending (LAD) artery, %	26	20	0.6096
Left circumflex (LCX) artery, %	4.3	1	0.5186
Right coronary artery (RCA), %	4.3	1	0.5186
LAD-LCX, %	41	20	0.0314*
LAD-RCA, %	8.6	2.1	0.2464
LAD-LCX-RCA, %	13.6	4.3	0.143

MINOCA: myocardial infarction with non-obstructive coronary arteries; LAD-LCX: left anterior descending-left circumflex.

* Statistically significant.

- Myocardial bridge (MB) was the most common cause of MINOCA (11.2%).
- The left anterior descending (LAD) artery was identified as the most common vessel responsible for ACS (26%).
- In cases with multiple vessel involvement, LAD-left circumflex (LCX) arterial involvement was the most frequent (41%).

## DISCUSSION

This study found that 6.28% of ACS patients had MINOCA, which is consistent with the MINOCA-TR study that reported a prevalence of 6.7%^10^. Other global studies, such as the ACTION Registry-GWTG and the ANZACS-QI program, have reported MINOCA rates ranging from 5.9 to 10.8%^11,12^. The variation in MINOCA prevalence may be attributed to differences in diagnostic criteria, population characteristics, and geographical variations. The lower prevalence in our cohort could reflect a selective sampling of ACS patients, and regional factors may influence these outcomes. Furthermore, it is crucial to recognize that MINOCA represents a complex clinical entity with varying etiology, which may not always be captured accurately by coronary angiography alone.

MINOCA patients in our study were predominantly male (83%) with a mean age of 53.9 years. This finding aligns with the existing literature, which demonstrates that young and male patients are more frequently diagnosed with MINOCA^4,10^. Additionally, the high rate of smoking (70%) in MINOCA patients in our study is an important risk factor. Studies have consistently shown that smoking not only contributes to the development of obstructive CAD but also plays a role in the pathogenesis of MINOCA^4,13^.

Other comorbidities, such as hypertension (45%) and diabetes mellitus (33%), were prevalent in our cohort, further emphasizing the need for prevention and management strategies targeting these risk factors. The higher incidence of hypertension and diabetes in MINOCA patients is in line with findings from other studies, where these conditions have been identified as contributing factors to myocardial injury, even in the absence of significant coronary stenosis^10,14^. Hypertension is associated with increased vascular stiffness, which can exacerbate myocardial Ischemia, even without significant coronary lesions. Similarly, diabetes mellitus impairs endothelial function and promotes inflammation, both of which may contribute to the development of MINOCA^10^.

Our study demonstrated that vitamin D levels were statistically significantly lower in MINOCA patients compared to controls (11.58±1.55 ng/mL vs. 33.29±3.28 ng/mL, p<0.001). This finding supports previous studies that have highlighted the role of vitamin D deficiency in vascular health and its association with CAD and acute ischemic events^15^. Vitamin D has anti-inflammatory properties, promotes endothelial function, and plays a role in vascular smooth muscle relaxation, which may help prevent ischemic injury^15,16^. Deficiency of vitamin D is also linked to arterial stiffness and vascular calcification, which are risk factors for coronary events, even in the absence of obstructive disease^15^. Furthermore, low B12 and folate levels in MINOCA patients may be indicative of the underlying oxidative stress and endothelial dysfunction, which are key contributors to ischemic injury in the coronary microcirculation^2,13^.

These findings suggest that assessing vitamin D levels and addressing nutritional deficiencies could be important in the management of MINOCA, especially in patients at risk of endothelial dysfunction and vascular complications. Future prospective studies focusing on supplementation with these nutrients could further elucidate their role in preventing MINOCA-related events.

MINOCA is a heterogeneous condition with multiple potential underlying causes. In our cohort, the most common cause of MINOCA was MB, which accounted for 11.2% of cases. This result is consistent with findings from other studies, which have shown that MB is a frequent cause of ischemic events, even in the absence of obstructive CAD^4,13^. MB occurs when the coronary artery is compressed during systole, leading to myocardial Ischemia. This phenomenon can result in ST-segment elevation, mimicking the presentation of an acute MI^4^. In addition to MB, we observed that coronary dissection, coronary ectasia, and hypertrophic dilated cardiomyopathy contributed to MINOCA in a small subset of patients. These conditions are known to cause microvascular dysfunction and transient Ischemia, which may not be detected by conventional angiography^4^.

Furthermore, the role of inflammatory triggers, such as cholangitis and pneumonia, was significant in our cohort. Inflammation has long been associated with endothelial dysfunction, which may predispose to Ischemia, even in the absence of coronary artery obstruction^13^. These findings support the hypothesis that systemic inflammation plays an important role in MINOCA pathogenesis and that anti-inflammatory therapies could be beneficial in the management of these patients.

In terms of coronary artery involvement, the LAD artery was the most common artery responsible for MINOCA (26%). Interestingly, when multiple coronary arteries were involved, LAD-LCX was the most common combination (41%). This suggests that coronary abnormalities affecting smaller coronary branches and the microcirculation may be more common in MINOCA patients than previously thought. The absence of significant coronary stenosis may mask the underlying pathology, which highlights the importance of using advanced imaging techniques, such as optical coherence tomography (OCT) and intravascular ultrasound (IVUS), to better characterize plaque morphology and the presence of vulnerable plaques^4,17^.

The proper management of MINOCA patients requires identifying the underlying pathophysiology. CMRI is a non-invasive diagnostic tool that can help identify causes, such as myocarditis, Takotsubo syndrome, and microvascular dysfunction^14^. In our cohort, CMRI could have provided additional insights into myocardial injury, especially in patients with suspected microvascular causes. Advanced imaging modalities like OCT and IVUS are essential in detecting vulnerable plaques, even when coronary angiography appears normal^13,17^.

As MINOCA is associated with significant morbidity and potentially poor outcomes, addressing modifiable risk factors, such as smoking cessation and hypertension management, is critical^4^. Given the high prevalence of smoking in our cohort, we recommend the widespread use of smoking cessation programs to reduce the risk of MINOCA and other cardiovascular events. Additionally, managing hypertension and implementing lifestyle changes such as increased physical activity and dietary modifications can help reduce the burden of endothelial dysfunction and inflammation, ultimately improving patient outcomes^4,14^.

### Limitations

This study provides valuable insights into MINOCA; however, it has several limitations. The retrospective design may have introduced bias, including missing data or incomplete patient histories. The absence of advanced imaging techniques, such as OCT and IVUS, may have led to an underestimation of the role of vulnerable plaques or microvascular abnormalities. As a single-center study, the findings are limited in terms of generalizability. Additionally, the lack of long-term follow-up data on mortality and recurrent MI restricts the understanding of the prognosis for MINOCA patients. Finally, the study did not fully account for potential confounding factors, such as genetic predispositions or environmental influences that could affect MINOCA risk.

## CONCLUSION

Key findings from this study highlight the role of modifiable risk factors, such as smoking, hypertension, and vitamin D deficiency, in disease management. MB was the most commonly identified cause, and vitamin D deficiency was also significantly associated with MINOCA. Further prospective studies with advanced imaging techniques and long-term follow-up are needed to better understand the pathogenesis and outcomes of MINOCA.

## Data Availability

The datasets generated and/or analyzed during the current study are available from the corresponding author upon reasonable request.
